# Behavioral and Cognitive Strategies in Pediatric Dentistry: Current Concepts in Anxiety and Fear Management

**DOI:** 10.7759/cureus.106784

**Published:** 2026-04-10

**Authors:** Hend Ahmed Alfadhli, Queen Deka, Indranil Samanta, Sneha Padmakarrao Masne, Priyanka Tripathi, Priti Sriranjan

**Affiliations:** 1 Department of Pediatric Dentistry, Batterjee Medical College, Jeddah, SAU; 2 Department of Psychology, University of Science and Technology, Baridua, IND; 3 Department of Community Medicine, Armed Forces Medical Service, New Delhi, IND; 4 Department of Oral Pathology and Microbiology, Bharati Vidyapeeth Dental College and Hospital, Navi Mumbai, IND; 5 Department of Dentistry, All India Institute of Medical Sciences, Gorakhpur, IND; 6 Department of Psychology, Narasingh Choudhury (Autonomous) College, Jaipur, IND

**Keywords:** behavior guidance, cognitive-behavioral therapy, dental anxiety, pediatric dentistry, sedation

## Abstract

Pediatric dental anxiety remains a prevalent and clinically significant barrier to effective oral healthcare, affecting treatment compliance, pain perception, and long-term oral health behaviors. Early negative dental experiences can consolidate maladaptive fear responses through neurobiological stress pathways and associative learning mechanisms, contributing to persistent avoidance and increased disease burden. This narrative review synthesizes contemporary evidence on behavioral and cognitive strategies for anxiety management in pediatric dentistry, integrating psychological theory, clinical frameworks, and emerging innovations. Core non-pharmacological approaches, including Tell-Show-Do (TSD), positive reinforcement, modeling, graded exposure, cognitive restructuring, relaxation training, and trauma-informed communication, are examined in relation to developmental stages, parental influence, and neurobiological underpinnings of fear regulation. The role of family-centered interventions and culturally responsive communication in strengthening therapeutic alliance and emotional security is highlighted. Pharmacological adjuncts, particularly nitrous oxide sedation and selected oral anxiolytics, are discussed as complementary modalities when behavioral strategies alone are insufficient. Emphasis is placed on integrative models that prioritize skill acquisition, emotional resilience, and gradual reduction in pharmacological reliance. Despite heterogeneity in study designs and outcome measures, current evidence supports child-centered, psychologically informed care as the cornerstone of sustainable anxiety management. Advancing standardized assessment tools and longitudinal research will further refine individualized, ethically grounded approaches in modern pediatric dentistry.

## Introduction and background

Pediatric oral health is significantly affected by dental anxiety, which commonly develops during the preschool and early school-age years and affects a substantial proportion of the pediatric population [1]. This condition extends beyond transient fear and includes emotional distress, physiological arousal, and uncooperative behavior, which can interfere with examination, prevention, and treatment. Repeated negative experiences may lead to dental avoidance, resulting in irregular attendance, increased caries risk, and poorer oral health-related quality of life over time [2]. Pediatric dental anxiety is multifactorial, involving biological, psychological, familial, and sociocultural influences. Neurobiological mechanisms, including activation of the amygdala and hypothalamic-pituitary-adrenal (HPA) axis, contribute to physiological stress responses such as increased heart rate and muscle tension [3]. These responses may reinforce fear memory and contribute to anticipatory anxiety. Parental attitudes and communication patterns also play a critical role, as negative expectations can be transmitted directly or indirectly to the child [4].

Contemporary pediatric dentistry emphasizes a child-centered, psychologically informed approach to anxiety management. Behavior guidance techniques such as Tell-Show-Do (TSD), positive reinforcement, modeling, and desensitization are widely used foundational strategies [5]. Cognitive-based interventions, including components of cognitive-behavioral therapy (CBT), aim to modify maladaptive thoughts, promote gradual exposure, and enhance self-regulation through techniques such as relaxation and guided imagery [6]. Family involvement is also essential, as caregiver behavior and communication influence children's responses; structured guidance can support positive modeling and reduce the transmission of fear [7]. Trauma-informed care and cultural competence further enhance anxiety management by promoting predictability, effective communication, and sensitivity to individual beliefs and prior experiences. Pharmacological approaches, including nitrous oxide sedation and oral anxiolytics, remain useful in selected cases, particularly in children with severe anxiety or limited cooperation [8]. However, reliance on pharmacological methods alone may limit the development of long-term coping skills. A balanced approach integrating behavioral, cognitive, and pharmacological strategies may optimize both immediate treatment outcomes and long-term psychological adaptation [9].

Despite these advances, challenges remain, including heterogeneity in anxiety measurement tools, variability in outcome reporting, and limited long-term data on behavioral change and reductions in pharmacological reliance. Additionally, children with neurodevelopmental disorders, trauma histories, or complex medical needs remain underrepresented in current research [10]. A comprehensive and integrated approach, combining insights from developmental science, behavioral psychology, and neurobiology, is required to enhance clinical outcomes. This review aims to synthesize current evidence on behavioral and cognitive strategies for managing pediatric dental anxiety, with emphasis on clinical applicability and underlying psychological mechanisms.

Objectives of the review

This review aims to critically synthesize the latest theoretical foundations and evidence-based strategies for managing dental anxiety in children, based on behavioral and cognitive therapies. Special attention is given to clinical applicability, the compatibility with the frameworks of family-centered and trauma-informed care, and the complementary role of pharmacological adjuncts in optimizing long-term emotional resilience and treatment cooperation.

Methodology

This narrative review was conducted following structured reporting principles aligned with PRISMA guidelines. A comprehensive literature search was performed using PubMed, Scopus, and Google Scholar to identify relevant studies published between January 2017 and January 2026. The search strategy combined keywords and Boolean operators, including: “pediatric dental anxiety” OR “dental fear in children” AND “behaviour guidance” OR “cognitive-behavioural therapy” OR “behaviour management” AND “sedation” OR “anxiety control in pediatric dentistry.” Reference lists of selected articles were also manually screened to identify additional relevant studies. Studies were included if they: (1) focused on pediatric populations (≤18 years), (2) evaluated behavioral, cognitive, or pharmacological approaches to dental anxiety management, and (3) were clinical studies, observational studies, or systematic reviews. Studies were excluded if they were non-English, case reports, conference abstracts, or not directly related to dental anxiety management in children.

Study selection was performed through initial screening of titles and abstracts, followed by full-text evaluation for eligibility based on predefined criteria. Relevant clinical guidelines and high-quality evidence-based studies were also included to enhance clinical applicability. Given the narrative nature of this review, a formal quantitative meta-analysis was not conducted. Instead, findings were synthesized using a thematic approach, grouping evidence into key domains, including epidemiology, behavioral strategies, communication, cognitive interventions, family-based approaches, and pharmacological adjuncts. A risk-of-bias assessment was not performed using a standardized tool due to the heterogeneity of included study designs; however, emphasis was placed on including peer-reviewed studies and higher-level evidence where available.

## Review

Epidemiology and pathophysiology of dental anxiety in children

Childhood dental anxiety is a common behavioral health issue that has significant consequences for oral health and access to healthcare. The prevalence differs among groups of children and adolescents, and it is linked to different factors, including gender, oral disease condition, and past dental experiences [11]. Regional variability is influenced by sociocultural factors, availability of preventive care, parental education, and prior experiences with invasive procedures. Avoidance behaviors, irregular dental visits, and the prevalence of untreated caries are linked with early-onset anxiety, which reinforces the cycle of fear and the development of disease [12]. Child-related, familial, and environmental determinants are multifactorial risk factors of pediatric dental anxiety. Behavioral inhibition, sensory sensitivity, and negative emotionality as temperamental factors predispose individuals to dental fear. Past traumatic dental or medical experiences, especially those related to pain or restraint, also contribute to anticipatory anxiety due to associative learning mechanisms [13]. Child behavior is affected by parental dental anxiety through modeling and verbal communication. Maladaptive fear reactions are also exacerbated by socioeconomic deprivation, lack of exposure to dental settings in early life, and irregular preventive dental treatment.

Dental anxiety is manifested at the neurobiological level as signs of central stress response system activity. The amygdala is activated by perceived threat stimuli and leads to a threat appraisal process and, secondarily, autonomic and endocrine reactions. This occurs through activation of the HPA axis, which leads to the release of cortisol and arousal of the sympathetic nervous system. Clinical symptoms include increased heart rate, elevated blood pressure, and muscle tension during dental procedures [14]. The consolidation of fear memories through encoding in the hippocampus strengthens a series of avoidance behaviors after repeated exposure to fear stimuli. Cognitive reappraisal may be constrained by immaturity of prefrontal cortical regulation in children, leading to heightened affective responsiveness. Dental anxiety is exhibited behaviorally according to developmental stages. Crying, clinging, tantrums, and physical resistance are typical of younger children, whereas withdrawal, somatic complaints, and decreased cooperation are more common in school-aged children [15]. Teenagers tend to develop anticipatory anxiety, catastrophic thinking, and concerns related to control and social perception. Identification of these patterns aids in providing behavioral guidance and early psychological intervention based on age. Table 1 shows the epidemiological distribution and pathophysiological mechanisms of dental anxiety in children.

**Table 1:** Epidemiological and pathophysiological correlates of pediatric dental anxiety HPA: hypothalamic-pituitary-adrenal

Domain	Key factors	Biological mechanisms	Clinical manifestations	Long-term impact	References
Global prevalence	10%-25% worldwide; higher prevalence in early childhood	Early stress sensitization	Avoidance of appointments	Irregular dental attendance	[4]
Temperamental risk	Behavioral inhibition and sensory sensitivity	Amygdala hyper-responsivity	Crying, clinging, and resistance	Persistent procedural fear	[7]
Trauma exposure	Painful prior procedures and medical restraints	Fear memory consolidation via the hippocampus	Anticipatory distress	Phobic reactions	[10]
Parental influence	Parental dental anxiety and negative modeling	Social learning pathways	Heightened vigilance	Intergenerational anxiety transmission	[1]
Neuroendocrine response	Perceived threat stimulus	HPA axis activation, cortisol release, and sympathetic arousal	Tachycardia, sweating, and muscle tension	Chronic stress reactivity	[5]

Psychological theories underpinning pediatric dental fear

Psychological models are used to explain the acquisition, maintenance, and expression of dental fear among children [16]. Classical conditioning describes the acquisition of fear through associative learning, where neutral stimuli are associated with painful or traumatic events. Conditioned fear responses are reinforced through repeated association between perceived stimuli, such as dental tools or noises, and distress. Operant conditioning also contributes to the persistence of anxiety, as avoidance behaviors are negatively reinforced when children evade or postpone dental care [17]. Social learning theory emphasizes the importance of observational learning in the development of dental phobia. Parental attitudes and verbal and nonverbal communication about dental experiences may be internalized by the child [9]. Fear can also be acquired vicariously through exposure to anxious peers or siblings and negative representations of the dental profession in the media, which influence anticipatory anxiety and maladaptive cognitive schemas [18].

**Figure 1:**
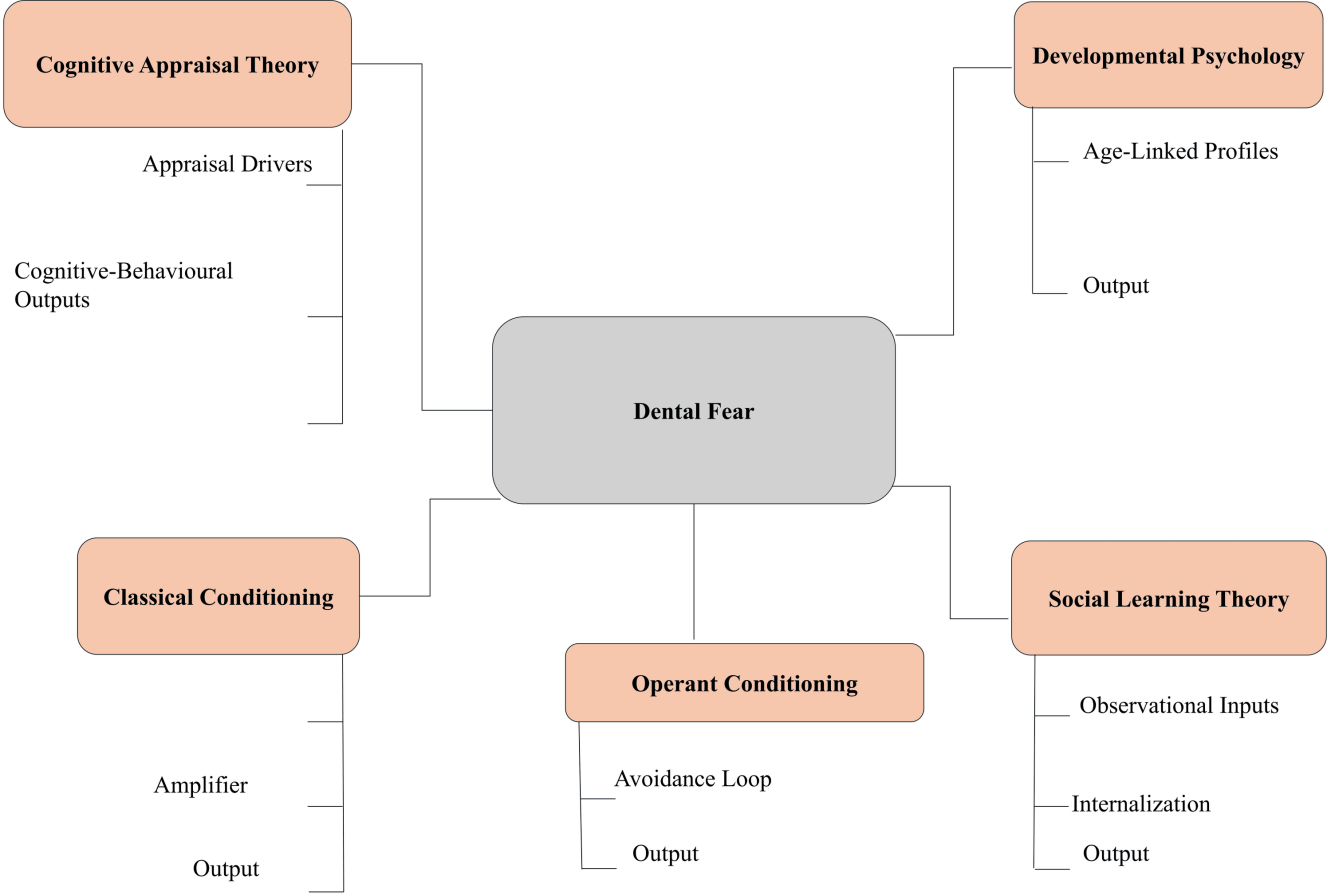
Psychological frameworks underlying pediatric dental fear Created by authors using Microsoft PowerPoint (Microsoft Corporation, Redmond, WA, US).

Cognitive appraisal theory focuses on the role of personal perception in the development of emotional reactions. Lack of perceived control, unfamiliarity with procedures, and overestimation of pain lead to heightened anxiety [11]. Children with poor coping abilities or distorted threat perceptions exhibit increased physiological arousal and behavioral resistance. Maladaptive thoughts, such as negative self-statements and overestimation of harm, also contribute to the exacerbation of anxiety responses in clinical contexts [19]. Developmental psychology accounts for differences in the expression of fear across age groups. Preschool children rely on concrete thinking and may have difficulty distinguishing between imagined and real threats, which increases procedural anxiety [20]. School-aged children develop a better understanding of causality but may remain sensitive to social evaluation and threats to physical integrity. Teenagers demonstrate more advanced cognitive processing, anticipatory rumination, and self-consciousness, which can further influence dental experiences [2]. A combination of these theoretical perspectives contributes to the development of customized behavioral interventions based on cognitive maturity and emotional regulation capacity. The psychological processes underlying the development and maintenance of dental fear are summarized in Figure 1.

Behavior guidance techniques in pediatric dentistry

The basis of non-pharmacological management of anxiety in pediatric dentistry is behavior guidance techniques. These strategies are used to foster cooperative behavior, improve emotional control, and develop trust within the clinical setting. The choice of a suitable technique is determined by the child’s developmental level, temperament, past experiences, and the complexity of the procedure [21]. One of the most widely accepted behavior guidance methods is TSD. It entails systematic explanation, simulation with non-threatening stimuli, and then implementation of the procedure. This approach enhances management of anticipatory anxiety through predictability and graded exposure, leading to cognitive assimilation of the dental experience [22]. Clinical evidence shows better compliance and less disruptive behavior with consistent and clear use of TSD.

Positive reinforcement is used to increase desirable behaviors through verbal praise or rewards, which enhance motivation and reinforce cooperative behavior [23]. Regular use leads to better compliance with appointments and long-term behavioral adaptation. Voice control involves adjusting tone, pace, and volume to redirect behavior and maintain attention. It helps maintain behavioral boundaries when used appropriately; however, ethical considerations require its careful use to avoid intimidation or emotional distress [8]. Facial expressions, eye contact, body language, and comforting touch are examples of nonverbal communication that are important for achieving psychological safety and building a stronger therapeutic relationship [24]. When nonverbal communication is congruent with verbal communication, children tend to respond better. Modeling involves exposure to cooperative peers or audiovisual demonstrations, promoting observation, strengthening coping expectations, and reducing uncertainty [25]. Desensitization involves progressive exposure to anxiety-causing stimuli, facilitating habituation and tolerance over time. Table 2 presents the main characteristics of these behavior guidance strategies.

**Table 2:** Core behavior guidance techniques in pediatric dentistry TSD: Tell-Show-Do

Technique	Core principle	Clinical application	Psychological basis	Expected outcome	References
TSD	Structured explanation and demonstration before the procedure	Demonstrating instruments before use	Gradual exposure and cognitive assimilation	Reduced anticipatory anxiety	[18]
Positive reinforcement	Rewarding cooperative behavior	Verbal praise and token rewards	Operant conditioning	Increased compliance	[12]
Voice control	Modulated tone to direct behavior	Calm, firm vocal guidance	Authority signaling and attentional focus	Behavioral redirection	[10]
Nonverbal communication	Supportive body language	Eye contact and reassuring gestures	Attachment theory: emotional attunement	Enhanced trust	[9]
Modeling	Observational learning from peers	Watching a cooperative child	Social learning theory	Improved coping expectancy	[11]
Desensitization	Stepwise exposure to stimuli	Gradual introduction of procedures	Habituation and fear extinction	Increased tolerance	[22]

Communication strategies and rapport building

Communication is one of the key determinants of behavioral outcomes in pediatric dentistry. Rapport building, emotional security, and treatment adherence are enhanced by developmentally appropriate interactions. Messages should be tailored to the child’s cognitive and linguistic development. Preschool children benefit from simple and concrete explanations, whereas school-aged children benefit from logical reasoning and discussion. Adolescents require communication that supports autonomy and privacy [26]. Threat perception largely depends on language framing. Anticipatory anxiety is alleviated through the use of age-appropriate, non-threatening language, which facilitates cognitive reframing of dental interventions. Avoidance of negatively connoted language helps prevent the triggering of fear-related schemas and catastrophic anticipations [7].

**Figure 2:**
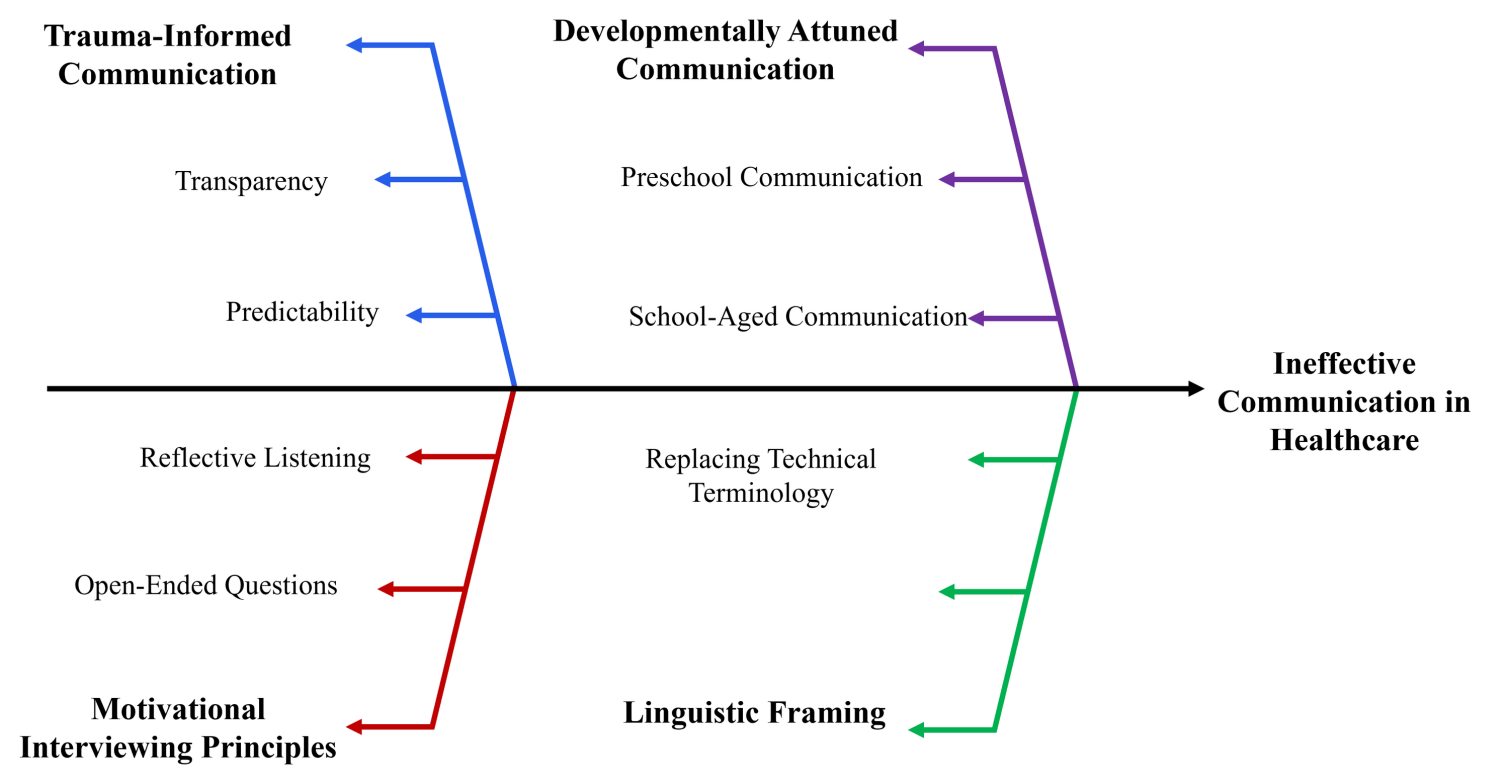
Structured communication strategies in pediatric dental care Created by authors using Microsoft PowerPoint (Microsoft Corporation, Redmond, WA, US).

Motivational interviewing techniques enhance engagement by promoting self-efficacy and shared decision-making. Open-ended questioning, reflective listening, and affirmation are strategies that facilitate behavioral change and reduce resistance during dental procedures [15]. Trauma-informed communication further enhances patient interaction by emphasizing predictability, transparency, and sensitivity to prior negative experiences. Children should be encouraged to express discomfort, and procedures should be explained in simple terms to improve perceived control and psychological safety [27]. Rapport building also requires cultural competence. Awareness of diverse health beliefs, language preferences, and family expectations promotes inclusive care and strengthens the therapeutic relationship. The use of culturally sensitive communication with caregivers enhances trust and continuity of care [28]. Procedural cooperation and emotional control, supported by developmental psychology and behaviorally informed structured communication strategies, enhance cooperation and reduce anxiety in pediatric dental settings. Figure 2 illustrates the core communication domains influencing cooperation and anxiety management in pediatric dental care.

Cognitive-behavioral therapy approaches in dental settings

CBT is an evidence-based approach to managing pediatric dental anxiety that focuses on maladaptive cognitions and behaviors [29]. It focuses on adjusting distorted perceptions of threats, improving coping mechanisms, and strengthening adaptive behavioral responses in clinical care. According to the cognitive model, dysfunctional interpretations of anticipated harm contribute to emotional distress. Exaggerated anticipation of pain and a perceived inability to control it increase physiological arousal and avoidance behavior in children [30]. One of the primary techniques is cognitive restructuring, which involves identifying and modifying irrational beliefs. Children are guided to replace catastrophic thoughts with more realistic and balanced ones, supported by encouragement and positive past experiences [31].

Guided imagery is used to refocus attention on calming mental representations, thereby reducing attention to procedural stimuli and lowering arousal levels [32]. Additional relaxation techniques, such as diaphragmatic breathing and brief muscle relaxation exercises, can help regulate physiological stress responses and enhance perceived control [33]. Exposure-based intervention involves introducing anxiety-provoking stimuli gradually, following a hierarchy, which facilitates habituation and the elimination of conditioned fear responses [34]. CBT techniques should be implemented chairside, taking into account time constraints and the child’s developmental level. Integrating cognitive restructuring, relaxation training, and graded exposure into routine dental care can enhance cooperation and contribute to long-term reductions in dental anxiety [35].

Parental and family-based interventions

Parental influence is an important factor in dental anxiety and behavioral reactions of pediatric patients undergoing dental treatment [21]. Caregivers can transmit dental fear to children through verbal and nonverbal communication, as well as behavioral modeling. Parents’ perceptions of threat can be internalized by children, leading to increased anticipatory anxiety and reduced cooperation through the observation of parental anxiety [36]. This process represents intergenerational transmission, which is reinforced by mechanisms of observational learning; even before direct dental experiences, expectations may be formed. The presence of parents during dental procedures should be determined on a case-by-case basis. Caregiver proximity may enhance emotional security and help regulate behavior in younger children. However, in some instances, parental anxiety or overprotective behavior can increase child distress and interfere with behavioral guidance techniques [37]. Decisions regarding parental presence should consider the child’s temperament, developmental level, and the caregiver’s emotional state.

**Figure 3:**
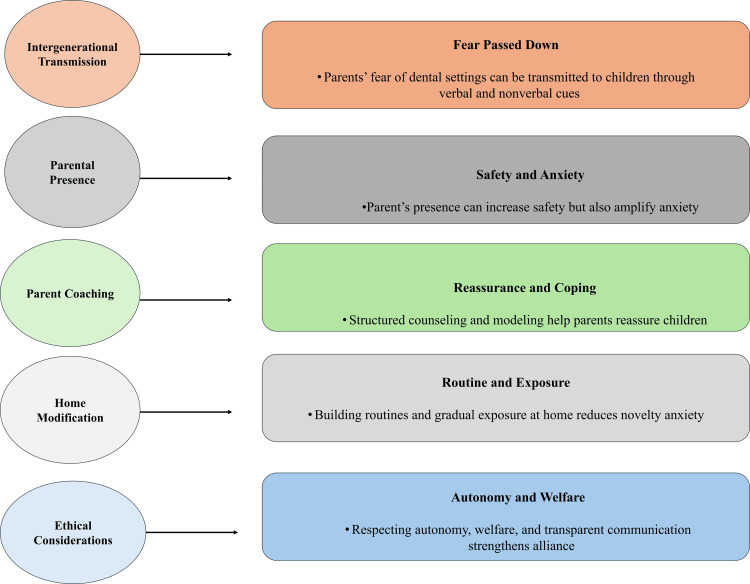
Family-centred determinants of pediatric dental anxiety and behavioural outcomes Created by authors using Microsoft PowerPoint (Microsoft Corporation, Redmond, WA, US).

Strategies to enhance child behavior can be effectively implemented by coaching parents. Caregivers can be trained in supportive communication methods, such as calm verbal interactions, appropriate facial expressions, and avoiding fear-inducing language [38]. Positive coping statements should be encouraged, and adaptive behaviors reinforced to facilitate emotional regulation. Pre-appointment counseling enables caregivers to adopt a calm and supportive role in the clinical environment. Family-based interventions extend beyond the dental clinic. Establishing home-based oral health routines can reduce anxiety associated with unfamiliarity. Desensitization can be promoted through gradual exposure to dental-related activities, such as practicing mouth opening or engaging in simulated dental procedures [39]. Home-based positive reinforcement further enhances cooperative behavior during dental visits. Family-centered care requires an ethical framework that balances parental involvement with the child’s psychological well-being. Elements such as transparency, informed consent, and cultural sensitivity are essential for maintaining trust and a strong therapeutic alliance [40]. The combination of family-based approaches contributes to improved emotional safety, procedural cooperation, and sustained involvement in oral healthcare. Figure 3 illustrates family-centered mechanisms influencing pediatric dental anxiety and behavioral responses.

Pharmacological adjuncts and their relationship to behavioral management

Pediatric dental anxiety is managed with pharmacological adjuncts that play a supportive role during the treatment process, particularly in cases of severe fear, low coping ability, or emergency situations [41]. Behavioral interventions are generally preferred, with pharmacological approaches employed at specific clinical points. Minimal sedation can enhance cooperation when used appropriately while maintaining psychological development [42]. The most frequently used pharmacological adjunct in pediatric dentistry is nitrous oxide-oxygen inhalation sedation due to its rapid onset, titratability, and favorable safety profile [43]. Its anxiolytic and mild analgesic effects reduce physiological arousal and increase receptiveness to behavioral guidance techniques. Maintenance of protective reflexes and verbal responsiveness allows communication-based interventions to be implemented simultaneously [44]. Oral sedation may be considered when anxiety is moderate to severe or when cooperation is insufficient. Midazolam and similar agents are anxiolytic, sedative, and partially amnesia-inducing. Proper use must be carefully evaluated based on medical history, weight-based dosing, and adherence to monitoring protocols [45]. Although oral sedation reduces acute distress, reliance on it without concurrent behavioral support may impair the development of coping skills in the long term.

There is a difference between pharmacological and behavioral approaches. Behavioral methods focus on cognitive appraisal, emotional regulation, and adaptive learning, whereas the use of sedation aims to balance neurochemical pathways and alleviate anxiety and discomfort. The best clinical practice is the continuance of behavioral involvement and pharmacological intervention to facilitate good experiences at the dentist [12]. Pharmacological interventions especially apply where behavioral strategies are insufficient; these are cases like children who require urgent treatment, those with neurodevelopmental disorders, and those with a history of trauma. Their application requires informed consent and observance of safety measures, as well as continuous monitoring of vital signs [23]. A more integrative treatment that incorporates low-level pharmacological intervention and behavioral therapy makes treatment easier and encourages the gradual development of coping skills. Gradual reduction of sedation between visits contributes to long-term resilience and autonomy. Pharmacological adjuncts and their integration with behavioral management strategies in pediatric dentistry are summarized in Table 3.

**Table 3:** Pharmacological adjuncts in relation to behavioral management N_2_O: nitrous oxide, O_2_: oxygen, CNS: central nervous system, MDZ: midazolam, GABA: gamma-aminobutyric acid, TSD: Tell-Show-Do

Modality	Mechanism of action	Clinical indications	Behavioral integration	Advantages and limitations	References
N_2_O-O_2 i_nhalation sedation	CNS depression, anxiolysis, and mild analgesia	Mild-moderate anxiety	Facilitates TSD and reinforcement	Rapid onset, minimal recovery time, and limited efficacy in severe fear	[32]
Oral sedation (e.g., MDZ)	GABA receptor modulation; sedation and amnesia	Moderate-severe anxiety	Requires concurrent behavioral guidance	Effective anxiolysis, variable onset, and monitoring essential	[28]
Conscious sedation	Controlled depressed consciousness	Complex procedures	Behavioral cues maintained	Preserves responsiveness and requires trained personnel	[34]
Behavioral techniques alone	Cognitive and emotional modulation	Mild anxiety	Primary modality	Promotes long-term coping and may be insufficient in extreme distress	[41]
Integrative approach	Combined pharmacological and behavioral modulation	High anxiety with treatment needs	Sequential skill reinforcement	Balanced efficacy and gradual reduction of sedation dependence	[38]

Special populations and individualized anxiety management

Pediatric dental anxiety management must be adapted for children with neurodevelopmental, medical, or psychological vulnerabilities [46]. Traditional behavioral interventions might not be effective in such groups, as they may have distorted sensory processing, communication difficulties, or increased responses to stress. Autistic children are known to have sensory hypersensitivity, difficulties with transitions, and a preference for routine. Visual schedules, predictable sequencing of procedures, and desensitization visits are strategies that enhance cooperation and minimize behavioral escalation [47]. Control is further facilitated by modifying the clinical environment to reduce sensory stimuli. Attention-deficit/hyperactivity disorder is common in children and is characterized by impulsivity, low attention span, and high activity levels. Strategies such as short appointments, planned activities, and immediate reinforcement can be used to maintain engagement. Instructions should be clear, and behavioral cues should be maintained to aid in task completion in the clinical setting [48].

Children with intellectual disabilities may be deficient in their understanding and adaptive functioning abilities. Providing simpler explanations, repetition, modeling, and involvement of caregivers are known to improve understanding and tolerance of the procedure. Progressive exposure is still critical for anxiety reduction and enhancing cooperation [49]. Children with complex medical histories are very likely to experience cumulative healthcare-related stress, which makes them more vulnerable to dental phobias. Psychological and physiological stability is encouraged by trauma-informed communication, explaining procedures, and improved monitoring [28]. Children who have experienced previous psychological or physical trauma need delicate and involved communication, with an emphasis on control, boundaries, and avoiding pressuring strategies. Developmentally relevant, individually tailored behavioral interventions maximize the results in a wide range of pediatric patients.

Limitations and future directions

The limitation of this review is that it depends on previously published literature and the heterogeneity of study designs, outcome measurements, and tools for assessing anxiety. Differences in age groups, cultural settings, and clinical settings could limit the generalizability of synthesized conclusions. Disagreements in methodological rigor between studies, such as small sample sizes and brief follow-up periods, decrease the power of comparative analysis. The interpretation of the effectiveness of some behavioral or pharmacological strategies may be affected by publication bias and underreporting of negative outcomes. There is a lack of longitudinal data, which limits the measurement of enduring anxiety outcomes and behavioral adaptation.

Standardized anxiety measurement tools and equivalent reporting standards should be the focus of future research to improve comparability. The durability of behavioral and cognitive interventions needs to be assessed in large-scale, multicenter trials with long follow-up periods. Integration of neurobiological data with digital monitoring devices could enhance early risk prediction. The sequencing of treatment could be clarified by comparative effectiveness studies on the use of combined behavioral and minimal pharmacological strategies. A higher degree of inclusion of diverse populations and special-needs cohorts will enhance external validity and inform individualized and culturally responsive clinical frameworks.

## Conclusions

This review finds that pediatric dental anxiety is multifactorial and significantly influences immediate treatment behavior as well as long-term oral health habits. Behavioral and cognitive interventions remain the primary strategies for reducing fear and improving cooperation. Developmental methods, such as TSD, positive reinforcement, graded exposure, cognitive restructuring, and relaxation training, have demonstrated consistent effectiveness in facilitating emotional regulation and adaptive coping. Communication patterns that emphasize empathy, predictability, and cultural sensitivity further enhance therapeutic relationships and patient confidence. Well-directed parental involvement also contributes to improved behavioral outcomes and long-term resilience. Pharmacological adjuncts are still useful in some situations where intense anxiety or special care requirements are involved, but combining them with behavioral support is a better practice when long-term skill development is desired. Individualized management pathways are continually being developed due to technological advances and trauma-informed care models. An ethically based, child-centered philosophy that incorporates both clinical and psychological understanding is the foundation of contemporary pediatric dentistry and contributes to the development of positive dental experiences and sustained reduction in anxiety.
